# Clinical Features and Patient Management of Lujo Hemorrhagic Fever

**DOI:** 10.1371/journal.pntd.0003233

**Published:** 2014-11-13

**Authors:** Nivesh H. Sewlall, Guy Richards, Adriano Duse, Robert Swanepoel, Janusz Paweska, Lucille Blumberg, Thu Ha Dinh, Daniel Bausch

**Affiliations:** 1 Internal Medicine, Morningside MediClinic, Johannesburg, South Africa; 2 Department of Medicine, University of the Witwatersrand, Johannesburg, South Africa; 3 Department of Medicine, University of Pretoria, Pretoria, South Africa; 4 National Institute of Communicable Disease, Sandringham, South Africa; 5 Centers for Disease control and Prevention, Atlanta, Georgia, United States of America; 6 Tulane School of Public Health and Tropical Medicine, New Orleans, Louisiana, United States of America; Children's Hospital Oakland Research Institute, United States of America

## Abstract

**Background:**

In 2008 a nosocomial outbreak of five cases of viral hemorrhagic fever due to a novel arenavirus, Lujo virus, occurred in Johannesburg, South Africa. Lujo virus is only the second pathogenic arenavirus, after Lassa virus, to be recognized in Africa and the first in over 40 years. Because of the remote, resource-poor, and often politically unstable regions where Lassa fever and other viral hemorrhagic fevers typically occur, there have been few opportunities to undertake in-depth study of their clinical manifestations, transmission dynamics, pathogenesis, or response to treatment options typically available in industrialized countries.

**Methods and Findings:**

We describe the clinical features of five cases of Lujo hemorrhagic fever and summarize their clinical management, as well as providing additional epidemiologic detail regarding the 2008 outbreak. Illness typically began with the abrupt onset of fever, malaise, headache, and myalgias followed successively by sore throat, chest pain, gastrointestinal symptoms, rash, minor hemorrhage, subconjunctival injection, and neck and facial swelling over the first week of illness. No major hemorrhage was noted. Neurological signs were sometimes seen in the late stages. Shock and multi-organ system failure, often with evidence of disseminated intravascular coagulopathy, ensued in the second week, with death in four of the five cases. Distinctive treatment components of the one surviving patient included rapid commencement of the antiviral drug ribavirin and administration of HMG-CoA reductase inhibitors (statins), N-acetylcysteine, and recombinant factor VIIa.

**Conclusions:**

Lujo virus causes a clinical syndrome remarkably similar to Lassa fever. Considering the high case-fatality and significant logistical impediments to controlled treatment efficacy trials for viral hemorrhagic fever, it is both logical and ethical to explore the use of the various compounds used in the treatment of the surviving case reported here in future outbreaks. Clinical observations should be systematically recorded to facilitate objective evaluation of treatment efficacy. Due to the risk of secondary transmission, viral hemorrhagic fever precautions should be implemented for all cases of Lujo virus infection, with specialized precautions to protect against aerosols when performing enhanced-risk procedures such as endotracheal intubation.

## Author Summary

Viral hemorrhagic fever is a syndrome often associated with high fatality and risk of secondary transmission. In 2008, an outbreak of a novel hemorrhagic fever virus called Lujo occurred in Johannesburg, South Africa, with secondary transmission from the index patient to four healthcare workers. Four of the five patients died. Lujo belongs to the arenavirus family and is only the second pathogenic arenavirus, after Lassa virus, to be recognized in Africa and the first in over 40 years. Because most viral hemorrhagic fevers occur in remote, resource-poor settings, few in-depth controlled studies of their clinical manifestations, transmission dynamics, pathogenesis, or response to treatment options are possible. We describe the clinical features of the five cases in this outbreak and summarize the clinical management, as well as providing additional epidemiologic detail. Lujo virus causes a clinical syndrome remarkably similar to Lassa fever. The treatment options used in these five cases are discussed as well as the recommended precautions to prevent secondary transmission.

## Introduction

Viral hemorrhagic fever (VHF) is an acute systemic illness classically involving fever, a constellation of initially nonspecific signs and symptoms, and a propensity for bleeding and shock.

VHF may be caused by more than 25 different viruses from four taxonomic families: *Arenaviridae*, *Filoviridae*, *Bunyaviridae*, and *Flaviviridae*. Transmission of hemorrhagic fever viruses is through direct contact with blood and bodily fluids during the acute illness. Although patient isolation and specific VHF precautions (consisting of surgical mask, double gloves, gown, protective apron, face shield, and shoe covers) are advised for added security, experience has shown that routine universal and contact precautions are protective in most cases [1]. Aerosol precautions, such as the use of N95 particulate filters, are only recommended when performing specific potentially aerosol-generating procedures, such as endotracheal intubation.

South Africa has often played a role of “sentinel” for VHF in countries further to the north through the travel and admission of undiagnosed patients to South African hospitals, often with subsequent nosocomial transmission to healthcare workers. For example, cases of Marburg and Ebola hemorrhagic fevers have been reported in Johannesburg in persons initiating travel in Zimbabwe [2] and Gabon [3], respectively.

**Figure 1:**
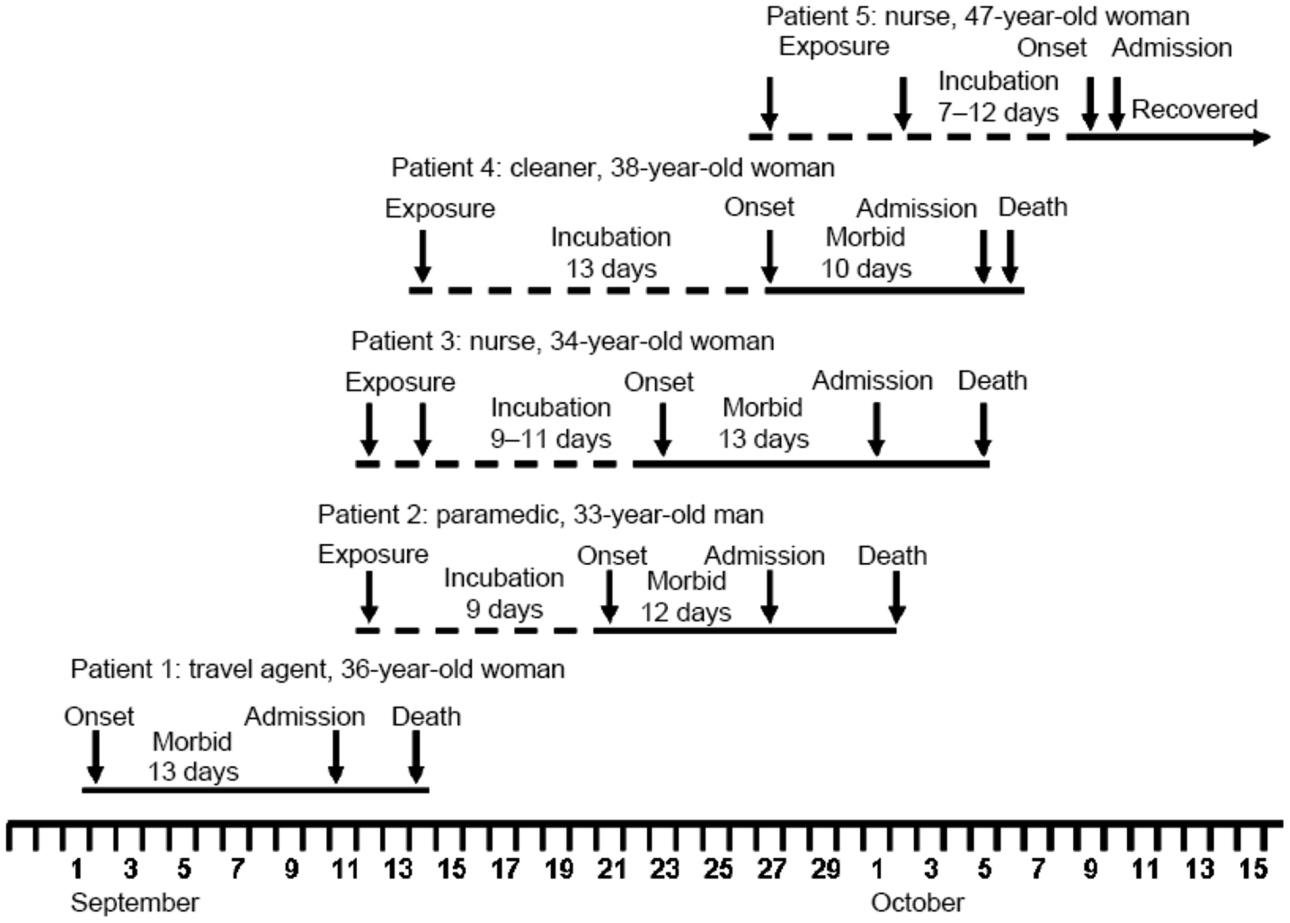
Timeline graph depiction of outbreak.

In 2008 a nosocomial outbreak of five cases of VHF occurred in Johannesburg [4], [5] (figure 1). The primary patient was a tour operator who was evacuated from Lusaka, Zambia. The etiologic agent was determined to be a novel arenavirus and the name “Lujo virus” was proposed. The source of the patient's infection is unknown, but assumed to be a rodent, as with all other pathogenic arenaviruses. Recent field studies of small mammals in Zambia did not result in isolation of Lujo virus, although another novel arenavirus was discovered [6].

Arenaviruses are divided into two groups: the New World (or Tacaribe) complex, and the Old World (or Lymphocytic Choriomeningitis/Lassa) complex, with various members of both groups causing VHF in South America and Africa, respectively [7] Lassa virus, the distribution of which is confined to West Africa, is the only other Old World arenavirus associated with VHF [8]. Lujo virus is only the second pathogenic arenavirus to be recognized in Africa and the first in over 40 years.

Some arenavirus infections, especially Lassa fever, have shown benefit with the use of the nucleoside analogue ribavirin [9]. Because of the remote and resource poor locations where Lassa fever typically occurs, as well as the history of civil unrest in West Africa in recent decades, there have been few opportunities to undertake in-depth study of the clinical manifestations or pathogenesis of Lassa fever or other VHFs, or the response of these infections to treatment options typically available in industrialized countries. We describe the clinical features of the five recognized cases of Lujo hemorrhagic fever (LHF) in the 2008 outbreak in South Africa and summarize their clinical management, as well as providing additional epidemiologic detail, with a focus on the risks for secondary transmission.

## Methods

### Ethics statement

The initial description of the outbreak [4] was published primarily under the auspices of the South African National Institute for Communicable Diseases, which had a blanket ethics approval for use of all the patients' data. The same data set has been used for this publication, with ethics committee approval, with the exception of further data collated on the one survivor, who provided written consent for use of data and images related to her illness.

### Case descriptions

#### Case 1

The initial case and primary patient (Patient 1) was a 36 year old white female who lived on the outskirts of suburban Lusaka, Zambia. She kept horses, dogs and cats at her house and evidence of rodents was found in her stables (Personal communication, R. Swanepoel). The patient fell sick on September 2 (Illness day [ID]-1) with the abrupt onset of fever, myalgia, sore throat, and headache, for which she took over-the-counter antipyretics and analgesics. The next day she described non-bloody diarrhea and vomiting. A mild erythematous rash appeared on ID-5 on her chest and upper arms. Fever up to 39°C continued intermittently, escalating on ID-7, accompanied by retrosternal chest pain and worsening sore throat, after which she presented to a clinic in Lusaka (ID-8), where she was given broad spectrum antibiotics. By ID-9 the rash covered her entire body. Myalgias became more prominent and her face was noticeably swollen. Rapid deterioration occurred on ID-10 with progressive confusion and generalized tonic-clonic seizures. She was intubated with some difficulty using only succinylcholine and started on further antibiotics, including ceftriaxone, ciprofloxacin and ampicillin.

The patient was evacuated by air ambulance to a private tertiary care hospital in Johannesburg on September 12 (ID-11). The Glasgow Coma Score was 3/10, with contracted non-reactive pupils and absent corneal reflexes but no papilledema—findings consistent with transtentorial brain herniation syndrome and damage to the pontine tegmentum from diffuse cerebral edema. Generalized edema, including of the face and neck, was present. There was no visible hemorrhage. A fine macular rash was observed over her torso and legs. An eschar resembling a tick bite was visible on her right foot. Diffuse interstitial infiltrates with bibasal atelectasis was noted on chest radiography. The patient received a tentative diagnosis of tick bite fever (*Rickettsia africae*) and was started on intravenous (IV) cefepime, clarithromycin, and linezolid, along with lactated Ringers solution and dobutamine. Mechanical ventilation was continued (FiO_2_ 0.8; BIPAP 20/10 mmHg; rate 14). The P_A_O_2_/F_i_O_2_ ratio was 160.

**Figure 2:**
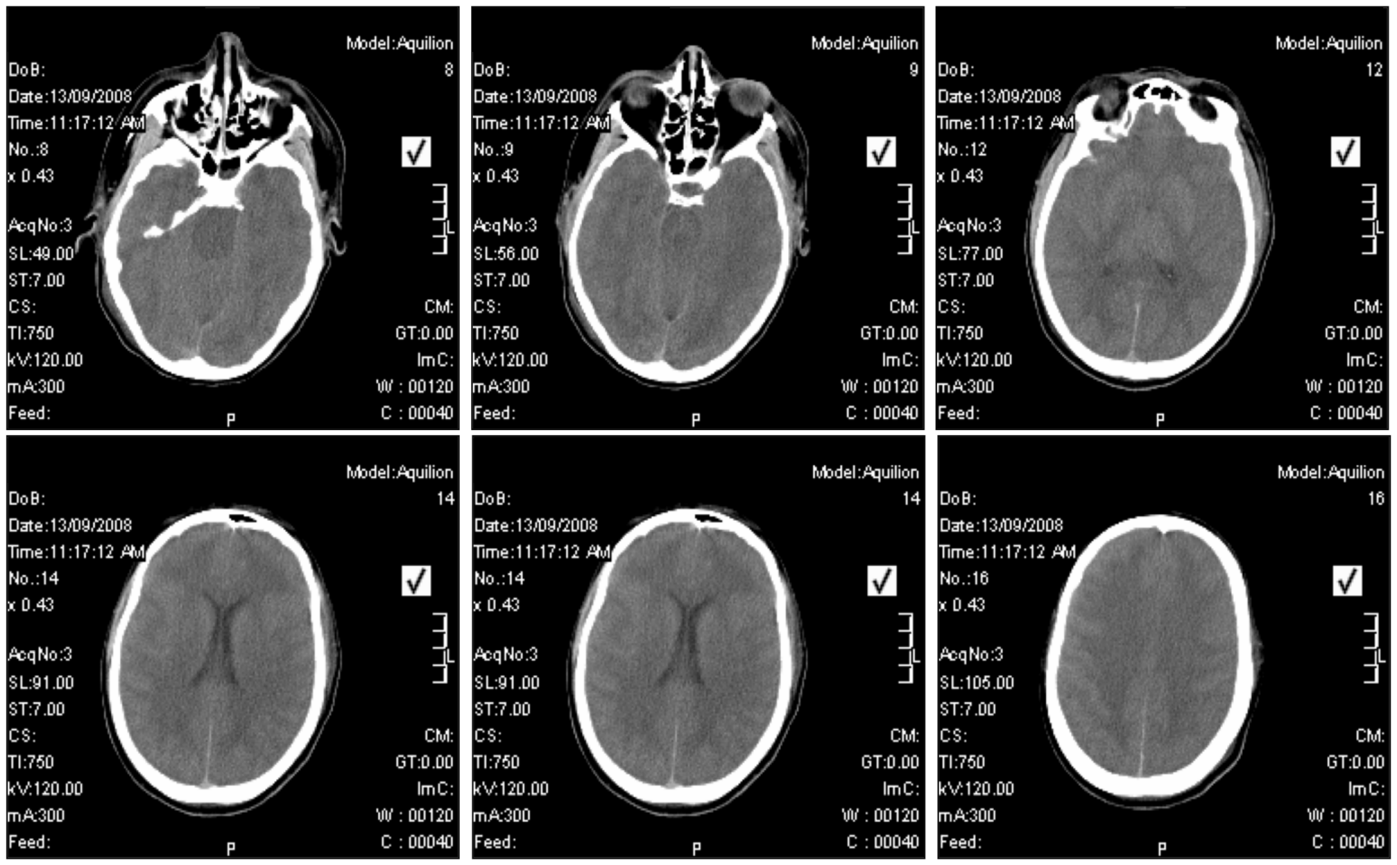
CT scan of the brain of Patient 5 showing cerebral edema on illness day 12.

On ID-12 progressive organ failure occurred. Oliguria was followed by a high anion gap metabolic acidosis and worsening generalized edema. Continuous veno-venous hemodialysis was commenced. A CT scan of the brain showed extensive cerebral edema with compression of the brainstem (Figure 2). An EEG showed diffuse slowing. Blood tests on ID-11 demonstrated leukocytosis (27×10^9^/L), thrombocytopenia (42×10^9^/L), elevated hepatic transaminases (AST 1,029 IU/L, ALT 386 IU/L) and lactate dehydrogenase (LDH 2,432 IU/L), and mildly elevated C reactive protein (CRP) (27 mg/L). The WBC rose to 58×10^9^/L the next day (ID-12). Blood cultures remained negative, as well as tests for malaria, typhoid fever, brucellosis, syphilis, and autoimmune disease. Rapidly progressive hemodynamic collapse and death occurred on ID-13 despite inotropic and vasopressor therapy.

#### Case 2

Patient 2, a 33 year old white male, was the paramedic who accompanied Patient 1 on the medical evacuation flight from Zambia to Johannesburg, subsequently returning to Lusaka. He participated in the intubation of Patient 1 at the referring hospital wearing disposable gloves but no gown, mask or face visor. No specific exposure to blood or other bodily fluids was noted.

On September 21 (ID-1), nine days after last contact with the index case, Patient 2 noted the abrupt onset of fever, headache and myalgias. Three days later (ID-4) he was admitted to a hospital in Lusaka for a possible upper respiratory infection and treated with oral amoxicillin and antipyretics. On ID-4 he developed a diffuse, erythematous skin rash, sore throat, and worsening myalgia and his fever rose to 40°C. Intravenous fluids and antibiotics were begun.

On ID-7 he was transferred to the same hospital in Johannesburg as Patient 1. Initial evaluation showed him to be fully awake and alert with a diffuse maculopapular eruption on his chest, arms, legs and back, sub-conjunctival hemorrhage, face and neck swelling, and pharyngitis, with ecchymoses on the hard and soft palates. He began to have non-bloody diarrhea. Clinical laboratory examination revealed thrombocytopenia (52,000/µL); leucopenia (2×10^8^/L); elevated transaminases (AST 969 IU/L, ALT 293 IU/L), LDH (2040 IU/L), and procalcitonin (2,0 ng/ml); marginally elevated CRP (27); a positive D dimer (>10 mg/ml); and microscopic hematuria. The INR was 1.42 and the partial thromboplastin time. (PTT) was elevated to 90 seconds. Tests for malaria, rickettsia, and salmonella were negative.

A presumptive diagnosis of thrombotic thrombocytopenic purpura was made and plasmapheresis initiated on ID-8. Prominent bleeding from the central vein insertion site was noted. On ID-9 the patient was seen by the intensive care unit (ICU) physician who cared for Patient 1 and an epidemiologic link was noticed. VHF precautions were immediately implemented. Given the history, possible filovirus infection was considered and contact tracing of the first patient was commenced by members of the hospital infection control team.

Modest improvement in Patient 2's condition was noted after plasmapheresis, with the platelet count increasing to 86,000/µL. However, rapid clinical deterioration began on ID-10, including altered mental status, oliguria, metabolic acidosis, and progressive generalized edema. Sustained low efficiency dialysis was begun and the patient was intubated due to worsening ARDS (P_A_O_2_/F_i_O_2_ ratio 100). Fulminant hepatitis (AST 3,763 IU/L; ALT 1,107 IU/L; LDH 7,207 IU/L), encephalopathy, and shock ensued and the patient died on ID-12 despite inotropic and vasopressor support.

#### Case 3

On October 2, the day of Patient 2's death, contact tracing revealed that an ICU nurse (Patient 3) who cared for Patient 1 was admitted to a private hospital west of Johannesburg, close to her family home. Patient 3 was a 34 year old black female who became ill on September 25 (ID-1), nine days after caring for Patient 1 (a previous publication on this outbreak erroneously cites this patient's first day of illness as September 23) [4]. She was primarily involved in turning and cleaning Patient 1, including washing the corpse and removing the dialysis catheter after her death. Infection control precautions in the care of Patient 1 included providing care in an isolation room and wearing of surgical gowns, latex gloves, surgical masks, and plastic visors. No needle stick injuries or splashes of blood or bodily fluids were reported.

Patient 3's illness began with headache and myalgia followed by sore throat, high fever, and rigors on ID-5. Oral amoxicillin and antipyretics were started by her general practitioner. Worsening headache and fever prompted hospitalization and isolation on ID-6, where nausea, abdominal cramps, non-bloody vomiting, and dysphagia were reported and a fine, macular rash noted on her trunk. (NB: Although Paweska *et al*. [4] reported that no rash was seen in the black patients with LHF, subsequent review of the treating physician's notes confirmed that a rash was indeed seen in this patient.) Clinical laboratory testing on admission was limited but demonstrated thrombocytopenia (78,000/µL) and normal transaminases (AST 18 IU/L, ALT 24 IU/L). Renal function was normal. Initial therapy consisted of IV fluids and ceftriaxone, fluconazole, and omeprazole.

The patient's condition worsened on ID-7 with non-bloody diarrhea, worsening rash, and peri-orbital and facial swelling. Sub-conjunctival hemorrhage was noted. Clinical laboratory analysis showed leukocytosis (13,000/µL), worsening thrombocytopenia (38.000/µL), and drastic elevations of liver enzymes (AST 2,182 IU/L; ALT 748 IU/L; LDH 3,421 IU/L). The quantitative D-Dimer was markedly elevated(>10.0 ug/l). Oral ribavirin (1,800 mg loading dose followed by 800 mg q8 hours) and IV gancyclovir (5 mg/kg q12 hr) were begun, the latter to cover the possibility of disseminated herpes virus infection. Nevertheless, the patient's condition worsened on ID-9, with continued diarrhea and facial edema, progressive mental obtundation, thrombocytopenia (47,000/µL) and persistently elevated transaminases (AST 2,486 IU/L; ALT 804 IU/L). A decision not to institute intensive care was taken collectively by the Provincial outbreak investigators given the circumstances at the time and the facilities available at the hospital. The patient became comatose and died on ID-10.

#### Case 4

Patient 4 was a 38 year old black female with a history of AIDS and a CD4 count of 250. She worked as a cleaner and was involved in the disinfection of the hospital room where Patient 1 died, which was performed wearing a scrub gown, surgical mask, plastic visor and surgical latex gloves. No specific exposures to blood or bodily fluids were reported.

Patient 4 fell ill on September 27 (ID-1), 13 days after cleaning Patient 1's room. Initial complaints included headache, dry cough, rhinitis, sore throat, myalgias and left sided chest pain. She visited her general primary care practitioner where a fever of 38.5°C was recorded and amoxicillin and diclofenac were prescribed. Five days later (ID-6), she presented to the infectious disease clinic at her local hospital. On the basis of fevers, sweating and an abnormal chest radiograph, outpatient therapy for tuberculosis was started. However, her condition continued to deteriorate and she was admitted to her local hospital on ID-8. At this point, the contact tracing team had located her and she was transferred to a tertiary academic hospital where she was noted to be confused with photophobia, nausea and vomiting. Physical exam showed candidiasis and generalized lymphadenopathy. Lumbar puncture and cerebrospinal fluid analysis showed five neutrophils and no lymphocytes, markedly elevated protein (>5 g/dl) and elevated glucose (7 mmol/L), which were considered consistent with a diagnosis of tuberculous meningitis. Clinical laboratory analysis revealed thrombocytopenia (23,000/µL), elevated transaminases (AST 549 IU/L, ALT 237 IU/L), mild renal dysfunction, a high anion gap metabolic acidosis, and a positive hepatitis B surface antigen. The patient's confusion worsened and fatal cardiac arrest occurred on ID-10.

#### Case 5

Patient 5 was a 47 year old white female who worked as an ICU nurse caring for Patient 2 from September 27–29. She had significant exposure to blood and bodily fluids, including cleaning up vomitus and changing bloody dressings over the insertion site of the central catheter on September 27. Although there was not yet a particular concern of VHF when the nurse was caring for Patient 2, she reported wearing plastic aprons, disposable gloves, and surgical masks, although she admits to potential lapses in the consistent wearing of this apparel.

Along with other contacts, Patient 5 was placed on twice daily temperature monitoring. On October 10 (ID-1), ten days after her last exposure to Patient 2, she noted a temperature of 38.4°C along with retro-orbital headache, nausea, and significant anxiety and was admitted to the hospital. Blood tests revealed thrombocytopenia (91,000/µL), leucopoenia (1,300/µL), normal levels of hepatic transaminases and an elevated D-dimer (2.84 µg/ml). A diagnosis of probable VHF was made (this was 2 days before an etiologic agent was identified). Since IV ribavirin was not available, oral ribavirin (2 g loading dose followed by 1 g q6 hrs) was begun on ID-2 along with atorvastatin (80 mg qd) and N-acetylcysteine (800 mg q8), both for their immunomodulatory and anti-inflammatory effects [10], [11], and anxiolytics.

**Figure 3:**
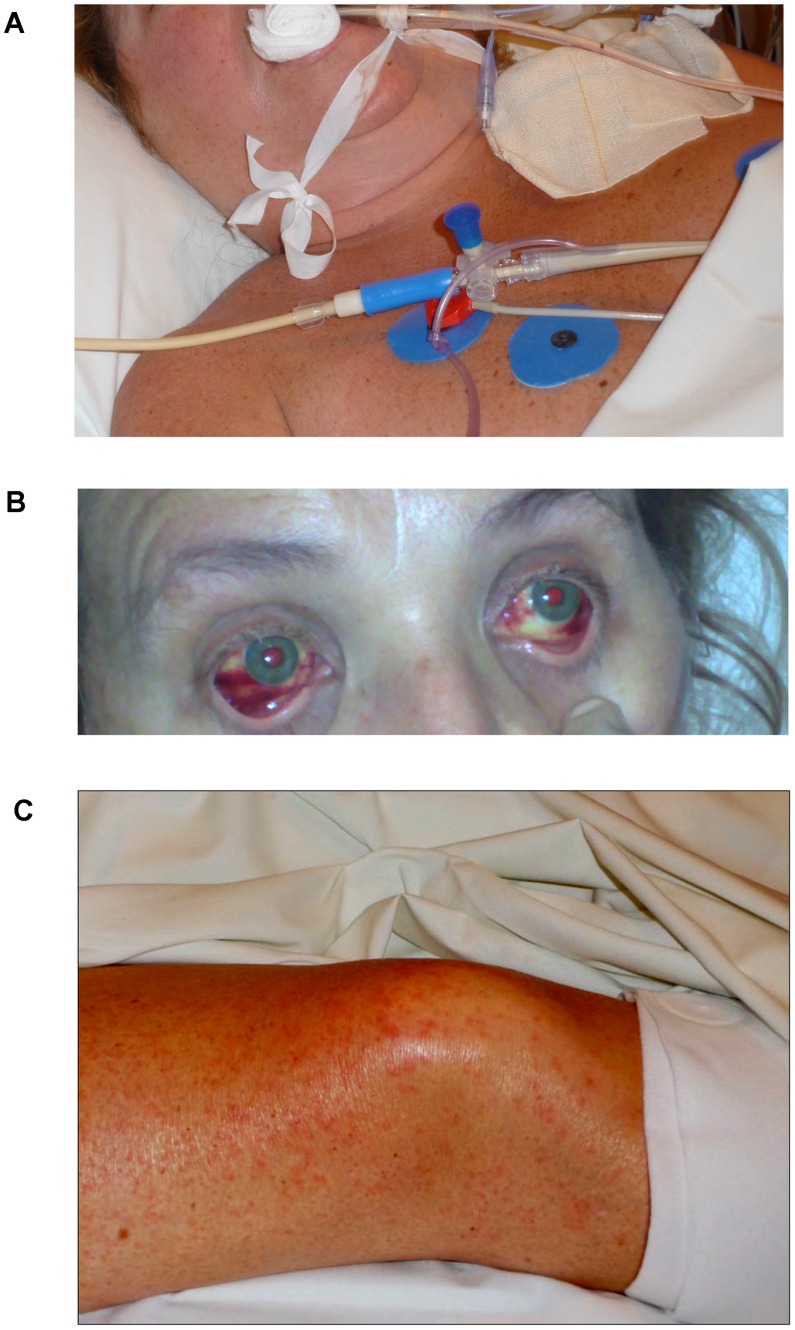
Clinical manifestations of Lujo haemorrhagic fever in Patient 5, including facial and neck swelling (A), subconjunctival haemorrhage (B), and maculopapular rash(C).

On ID-2 myalgias became prominent and thrombocytopenia worsened (61,000/µL). On ID-3 the temperature was 38.6°C and non-bloody diarrhea and vaginal bleeding began, despite the patient being midcycle. Laboratory tests on ID-4 show a leukocyte count of 7,100/µL, elevated transaminases (AST 192 IU/L, ALT 81 IU/L), and a prolonged PTT of 60 seconds (control 31 seconds). Drowsiness and exudative pharyngitis, including a peri-tonsillar pseudo-membrane, were present. On ID-5 the patient complained of odynophagia and facial edema and a resting tremor were noted (Figure 3A). Despite being clinically hypovolemic, relative bradycardia (HR 68/minute) was present. Thrombocytopenia (48,000/µL) and transaminitis (AST 209 IU/L, ALT 82 IU/L) worsened. Intravenous recombinant factor VIIa (1.2 mg q6 hrs) was begun and the N-acetylcysteine was switched to IV administration (1 g q8 hrs). On ID-6, the facial edema was slightly improved but palatal ecchymoses were noted along with conjunctival injection. To cover possible bacterial or fungal super-infection, IV cefepime (2 g q12 hrs) and fluconazole (400 mg q12 hrs) were started.

Non-bloody diarrhea and hypotension (BP 80/40 mmHg) with relative bradycardia (HR 64/min) persisted on ID-7. The patient became tachypnoeic, with basilar crackles noted on auscultation. A decision was made to intubate the patient but the procedure, although ultimately successful, proved difficult due to the swollen airway with a pseudo-membrane extending to the glottic folds. Multiple, coalescent hemorrhagic areas were present in the hypopharynx. Minor contact bleeding followed suctioning. A central line was inserted, with significant bleeding around the insertion site. Ribavirin was continued via naso-gastric tube until a supply of IV ribavirin was finally obtained and administered at 20 mg/kg q6 hrs in place of the oral drug.

The patient's condition improved on ID-8, with better hemodynamic parameters and reduction in mechanical ventilation (FiO_2_ 0.4). No focal neurologic deficits were noted on interruption of sedation. Subconjunctival hemorrhage was noted and a fine, blanching, erythematous, maculopapular rash appeared on her trunk, arms and legs, sparing the palms and soles (Figure 3B and 3C). The patient continued to steadily improve, with the nadir of thrombocytopenia on ID-12 (23,000/µL) coinciding also with the peak transaminase level (AST 235 IU/L, ALT 119 IU/L). The rash resolved by ID-14.

Distal neuropathic weakness appeared on ID-13 and hepatomegaly and splenomegaly on ID-15. She was weaned from mechanical ventilation on ID-15. However, on ID-17 she developed sinus tachycardia (130/min) associated over the following four days with basal crackles and an S3 gallop rhythm treated with diuretics and carvedilol. This finding was attributed to myocarditis, a conclusion supported by the finding of an elevated NT-pro BNP level (1000 pg/ml). On ID-18, the ten day course of IV ribavirin was completed and ribavirin, recombinant factor VIIa, and N-acetylcysteine were stopped. No further bleeding was noted. Thrombocytopenia improved (86,000/µL) and the AST was down to 177 IU/L, although the PTT was still elevated (50 sec) on ID-39. The Russell's viper test for lupus anticoagulant was weakly positive.

The patient recovered slowly and was discharged from hospital on November 2 (ID-42). Neurologic features were prominent during the patient's recovery. Anxiety, mood fluctuation, and confusion were considered consistent with post-traumatic stress disorder for which she was treated with antidepressants and anxiolytics, which were slowly weaned after one year. Distal critical illness peripheral neuropathy and myopathy, tremors, and weakness persisted for at least 6 months after hospital discharge. No hearing loss was noted, although formal audiometry was not performed.

Her sinus tachycardia resolved by ID-52. Complete non-scarring alopecia developed from ID-83 and resolved slowly over a four month period. Repeat tests for lupus anticoagulant were negative.

## Results

### Summary of cases

The five patients' ages ranged from 33 to 47 years. There were two white females, two black females, and one white male. The incubation periods of the 3 secondary and 1 tertiary cases ranged from 9-13 days. Four of the five patients died (CFR 80%).

#### Signs and symptoms

The signs and symptoms of the five patients are presented in Table 1. In all cases, the clinical illness began with the abrupt onset of common and nonspecific symptoms, including fever, malaise, headache, and myalgias, that would not particularly raise suspicion of VHF. Sore throat (in one case accompanied by pharyngeal exudates), non-bloody diarrhea, and nausea and vomiting readily ensued, sometimes accompanied by retrosternal or epigastric pain. A blanching erythematous maculopapular rash on the torso extending to the limbs, but sparing the palms and soles, appeared toward the end of the first week of illness in 4/5 patients and seemed to coalesce before fading and disappearing in the sole survivor by ID-14. Subconjunctival injection or hemorrhage and swelling of the face and neck appeared slightly after the rash in most cases, around the end of the first week of illness. Neurological signs were less frequent, but included tremors and seizures, the latter in the end stages of disease and accompanied by cerebral edema noted on CT scan. Hepatomegaly and splenomegaly developed in the survivor by ID-15 persisting until ID-40. No episodes of major hemorrhage were noted, although minor hemorrhage was common in the later stages of disease, including the aforementioned sub-conjunctival hemorrhage, palatal ecchymoses, and bleeding at injection sites. Rapid clinical deterioration consistent with shock and multi-organ system failure was noted between IDs 7–10, with death a mean of nine days (range 6–12 days) in the four fatalities. The Simplified Acute Physiology Score II (a predicted mortality score derived from measurement of various physiologic parameters 24 hours after ICU admission) for the four fatal cases ranged from 4.7% to 73%, compared to 28.5% for the surviving patient. Convalescence was protracted for the survivor.

**Table 1:** Clinical signs and symptoms in 5 patients with Lujo hemorrhagic fever.

Sign or symptom	# Manifesting	Mean day of illness in which sign or symptom first appeared (range)	Comments
Fever	5/5	1 (-)	Range 38.2–40°C
Headache	5/5	1 (-)	
Cough	1/5	1 (-)	Patient also had AIDS
Rhinitis	1/5	1 (-)	Patient also had AIDS
Myalgia	5/5	1.2 (1–2)	
Sore throat or pharyngitis	5/5	3.2 (1–6)	
Chest pain	2/5	4.0 (1–7)	
Nausea and/or vomiting	4/5	4.3 (2–8)	
Diarrhea	4/5	4.5 (2–7)	All diarrhea was non-bloody
Rash	4/5	5.8 (4–8)	Typically maculopapular, starting on the torso and spreading to the limbs
Oliguria	3/5	9.3 (7–11)	
Relative bradycardia	1/5	5 (-)	
Hemorrhage (excluding sub-conjunctival hemorrhage)	5/5	5.5 (3–8)	Includes vaginal bleeding (1/5, day 3), pharyngeal ecchymoses (2/5, days 6 and 7), and bleeding at central vein catheter insertion site (2/5, days 7 and 8). 4/5 patients had bleeding at injection sites.
Sub-conjunctival injection or hemorrhage	3/5	6.7 (6–7)	
Crackles on auscultation	1/5	7 (-)	
Facial and/or neck swelling	4/5	7.0 (5–9)	
Neurological signs	2/5	7.5 (5–10)	Includes tremor (1/5, day 5), seizures (1/5, day 10)
Photophobia	1/5	8 (-)	Patient also had AIDS
Lymphadenopathy	1/5	8 (-)	Patient also had AIDS

Signs and symptoms are listed in order of appearance during the course of infection. Only manifestations noted during the first 2 weeks of illness are shown.

#### Clinical laboratory findings

Clinical laboratory findings for the five patients are presented in Table 2. Typical findings included early leucopenia and lymphocytopenia followed later by leukocytosis, thrombocytopenia, and elevated LDH and transaminases, with AST generally 2–3 times greater than ALT. Elevated D-dimer levels and prolonged PTT consistent with disseminated intravascular coagulopathy (DIC) were noted in three patients. No red cell fragmentation was seen but microscopic hematuria was documented in 3/5 patients. Other notable laboratory results included mildly elevated BUN (3/5 patients) and creatinine (2/5 patients) and normal or slightly elevated levels of CRP and procalcitonin.

**Table 2:** Clinical laboratory parameters in 5 patients with Lujo hemorrhagic fever.

Laboratory Parameter	Reference range	Patient 1	Patient 2	Patient 3	Patient 4	Patient 5	Range for all patients
Hb (g/dl)-peak*	12.2–16.7	17.1(d12)	15.7(d9)	17.7(d10)	ND	14.8 (d5)	14.8-
Hb (g/dl)-admission	g/dl	15.2	14.2	13.0		14.8	17.7(d5–12)
Hb (g/dl)-nadir*		15.2 (d11)	13.7 (d8)	13.0 (d6)	ND	6.7 (d27)	6.7–15.2(d6–27)
HCT (%)-peak*	35–49%	41.0	42.0	38.0	ND	40.0	38–42
HCT (%)-admission		41	42	44	-	45	
HCT (%)-nadir*					ND		
WBC peak (x10^9^/ℓ)	4–12×10	80 (d13)	28 (d7)	25 (d10)	14 (d8)	18 (d5)	14–80 (d1–7)
WBC (x10^9^/ℓ)-admission		27	2.14	5.9	14.9	1.16	
WBC differential-peak (%) Abs(x 10^9^/l)							
Neutrophils		90 (72)	54 (15.12)	78 (19.5)	ND	59 (10.62)	54–90
Lymphocytes		5 (4)	41 (11.48)	20 (5)	ND	36 (6.4)	5–41
Eosinophils		2 (1.6)	1 (0.28)	0	ND	6.1 (1.09)	0–6.1
Basophils		0	0	0	ND	0.8 (0.14)	0–.8
WBC nadir (x10^9^/ℓ)	4–12×10	27 (d11)	2,1 (d8)	4,8 (d6)	14 (d8)	1,3 (d1)	1.3–27(d1–11)
WBC differential-nadir (%) Abs (x 10^9^/l)							
Neutrophils		90 (24.3)	54 (1.13)	78 (3.7)	ND	59 (0.76)	54–90
Lymphocytes		5 (1.35)	41 (0.86)	20 (0.96)	ND	36 (0.48)	5–41
Eosinophils		2 (0.54)	1 (0.02)	0	ND	6.1 (0.07)	0–6.1
Basophils		0	0	0	ND	0.8 (0.01)	0–8
Platelets (x10^9^/ℓ)-peak*	150–450×10	85 (d12)	121 (d7)	102 (d6)	23(d8)	91 (d1)	23–102(d1–12)
Platelets (x10^9^/ℓ)-admission		42	56	78	23	104	
Platelets (x10^9^/ℓ)-nadir*	150–450×10	42 (d10)	46 (d9)	38 (d8)	23 (d8)	23 (d6)	23–46(d6–10)
AST (iu/ℓ)-peak	13–35	1029 (d10)	3763 (d7)	2486 (d8)	549 (d8)	280 (d8)	280–3763(d7–10)
AST (iu/ℓ)-admission		1029	969	218	549	30	
ALT (iu/ℓ)-peak	<35	386 (d10)	1008 (d7)	804 (d9)	237 (d8)	156 (d17)	156–1008(d7–17)
ALT (iu/ℓ)-admission		386	293	24	237	19	
AST/ALT ratio		2,6	3,7	3,1	2,3	1,8	1.8–3.7
Albumin (g/l)-nadir	35–50	16 (d10)	25 (d7)	23 (d9)	23(d8)	10 (d6)	10–25(d6–10)
Albumin (g/l)-admission		16	33	42	23	47	
LDH (iu/ℓ)-peak	120–230	2432 (d10)	7207 (d7)	4540 (d9)		2069 (d16)	2069–7207(d7–16)
Bilirubin (umol/ℓ)-peak	2–26	5 (d10)	23 (d7)	26 (d9)		70 (d15)	5–70(d7–15)
Bilirubin (umol/ℓ)-admission		5	6	21	-	4	
CRP (mg/ℓ)-peak	<5	62 (d12)	35 (d8)	114 (d7)		78 (d8)	35–114(d7–12)
CRP (mg/ℓ)-admission		27	30	114	-	3.3	
Procalcitonin (ng/ml)-peak	<0.05	1,22 (d12)	2,09 (d8)				1.22–2.09(d8–12)
Procalcitonin (ng/ml)-admission		0.32	2.0	-	-		
D-Dimer (ug/ml)-peak	<0.5		>10 (d8)	>10 (d8)		2,8 (d1)	2.8–>10(d1–8)
D-Dimer (ug/ml)-admission		-	>10	-	-	2.84	
PTT (sec)-peak	27–43		68 (d8)	74 (d1)		40 (d1)	40–68(d1–8)
PTT (sec)-admission		-	90		-	40	
INR-peak	1–1.25	2,93 (d13)	1,12 (d9)	1,91 (d10)		1,2 (d35)	1.12–2.93(d9–35)
Calcium (corrected)(mmol/ℓ)-nadir	2.15–2.65	2,19 (d11)	1,84 (d9)			2,14 (d25)	1.84–2.19(d9–25)
Calcium (corrected)(mmol/ℓ)-admission		2.19	-	-	-		
Ammonia (umol/ℓ)-peak	16–60		149 (d7)				
Sodium (mmol/ℓ)-peak	135–150	141 (d13)	137 (d9)	140 (d10)	136 (d8)	134 (d1)	134–141(d1–13)
Sodium (mmol/ℓ)-admission		137	132	133	133	134	
Potassium (mmol/ℓ)-nadir	3.5–5.1	3.3(d13)	3.5(d7)	3.3(d10)	5.1(d8)	3.1(d3)	3.1–5.1(d3–13)
Potassium (mmol/ℓ)-admission		4.3	3.5	3.4	5.1	3.6	
Urea (mmol/ℓ)-peak	2.1–7.1	8,5	7,9	1,9	10,8	2,8	1.9–10.8
Urea (mmol/ℓ)-admission		2.8	5.6	1.6	10.8	2.8	
Creatinine (umol/ℓ)-peak	80–115	118.0	160.0	66.0	97.0	72.0	66–118
Creatinine (umol/ℓ)-admission		31	136	88	97	72	
Total CO_2_ (mmol/ℓ)-peak	21–29	17.0	20.0	19.0	9.0	25.0	9–25
Total CO_2_ (mmol/ℓ)-admission		24	22	28	9	25	
Estimated GFR (ml/min)-nadir	>60	48.0	43.0	>60		>60	43–>60
Estimated GFR (ml/min)-admission		>90	52	>60	-		
ESR-peak				2 (d6)			
Fibrinogen (g/ℓ)-nadir	2–4		2.09(d7)			5.04(d39)	2.09–5.04(d7–39)
Antithrombin III (%)-	87–140		109(d7)				
Russell's viper test(ratio, s)	1.24–1.50					1.61(d39)	

The day of illness that the value was noted is in parentheses.

*Patients 1, 2, and 5 received transfusions of packed red blood cells, platelets, and fresh frozen plasma during the course of their illnesses.

Abbreviations: ALT-alanine aminotransferase, AST-aspartate aminotransferase, CRP-C reactive protein, ESR-erythrocyte sedimentation rate, Hb-hemoglobin, HCT-hematocrit, INR-international normalized ratio, LDH-lactate dehydrogenase, ND-not done, PCT- procalcitonin, PTT-partial thromboplastin time, WBC-white blood cell count.

#### Clinical management

Although epidemiological links were made between many of the patients as the outbreak progressed, the diagnosis of arenavirus infection was not made until October 13 (ID-3 of Patient 5's illness). Furthermore, the five patients were hospitalized at three different centers in South Africa and treated by different healthcare workers. Thus, there was little opportunity for uniformity of clinical approach. Management of the non-survivors included IV fluids (4/4); broad spectrum antibiotics (4/4); transfusion of packed red blood cells, platelets, and fresh frozen plasma (2/4); hemodialysis (2/4); mechanical ventilation (2/4); plasmapheresis (1/4); and oral ribavirin (1/4, but the patient received only three doses before death). The surviving patient received many of these same treatments. Distinguishing characteristics of her care which could have played a role in her survival include rapid commencement of ribavirin (oral ribavirin was begun on ID-1 with conversion to IV on ID-8), and the administration of recombinant factor VIIa, N-acetylcysteine, and atorvastatin on ID 2.

## Discussion

Based on the five cases of LHF recognized to date, the clinical disease associated with LHF is remarkably similar to Lassa fever [7]. Surprisingly, the two viruses are genetically quite distinct (up to 38.1% on the nucleotide level), with Lujo virus grouping much closer genetically to Old World arenaviruses not associated with VHF [5] Lassa fever classically begins with non-specific signs and symptoms including fever, general malaise, headache, myalgia, chest or retrosternal pain, and sore throat with progressive diarrhea and other gastrointestinal involvement [7], [9]. Severe cases may progress to a capillary leak syndrome with septic shock, rash, facial and neck swelling, and multi-organ system failure. The facial and neck swelling seen in both LHF and Lassa fever appear to be specific to Old World arenavirus infection and may help differentiate it from other African VHFs. Like in Lassa fever (and despite the slight misnomer “VHF”), major bleeding was not a prominent feature in the patients with LHF, although minor bleeding was common. The AST and ALT are typically elevated in Lassa fever, with AST much greater than ALT and high levels of AST associated with a poor prognosis [7]. This same pattern was seen in all five patients with LHF, with the only survivor manifesting the lowest peak AST and AST: ALT ratio.

Some distinctive features of LHF relative to typical Lassa fever were the abrupt disease onset (typically indolent in Lassa fever) and the presence of DIC, which is generally not considered to be part of the pathogenesis of Lassa fever, although the matter has not been extensively studied [9]. Although rash is consistently seen in light-skinned persons with Lassa fever, for unknown reasons it is almost never seen in blacks. All of the white patients and one of the two black patients with LHF manifested a very prominent rash. Interestingly, the black patient without rash was HIV infected, suggesting that the rash of LHF may be immune mediated. Patient 5 also had relative bradycardia, an interesting finding given reports of depressed cardiac function in an animal model of arenavirus infection [12].

The CFR associated with this outbreak of LHF was 80%. The CFR of hospitalized patients with Lassa fever is typically in the 20–30% range, ranging up to 50% in some nosocomial outbreaks [13]. However, mild and asymptomatic Lassa virus infection is thought to be common, with mortality rates less than 5% when infection in the community is considered [7], [14]. No antibody survey of case contacts or community members in the region of origin of the index case in Zambia has been conducted to determine if mild or asymptomatic infection with Lujo virus occurs.

The four nosocomial infections of Lujo virus illustrate the risk to healthcare workers. Although no specific exposures were reported and some degree of personal protective equipment was worn by all four secondary or tertiary cases, it appears that strict barrier nursing practices were not always maintained and full VHF precautions were often implemented late in the course of treatment, if at all. Furthermore, the four infected healthcare workers generally had very close and sometimes prolonged contact with the patient, including in closed settings, such as the medical evacuation flight of Patient 1, augmenting the possibility of exposure to blood and bodily fluids. They also performed procedures that are often considered to be high risk, such as endotracheal intubation, insertion of indwelling intravascular catheters, and dialysis. The transmissibility of other emerging viruses such as SARS and MERS coronaviruses has similarly been enhanced when such procedures have been performed [15]. In addition to the 4 secondary/tertiary cases, another 94 persons were identified as contacts and monitored, including support staff (kitchen, laundry, cleaning), laboratory and radiography technicians, and nursing staff. We did not categorize contacts in terms of risk at the time, but now estimate that at least 30 of these would be reasonably categorized as high risk. Nevertheless, no suspected cases of LHF were noted in this group.

We suspect that the degree of transmissibility of Lujo virus is likely analogous to that of Lassa virus, for which, although reliable reproduction numbers and secondary attack rates are difficult to ascertain, they are generally thought to be low. Nevertheless, occasional outbreaks with secondary and tertiary cases are sometimes seen, especially when barrier nursing practices are not maintained [16], [17]. Until the matter can be studied more thoroughly, VHF precautions should certainly be implemented for all suspected and confirmed cases of LHF, with specialized precautions to protect against aerosols when performing endotracheal intubation [1].

Despite the high prevalence of HIV infection in many areas of sub-Saharan Africa, including some areas where VHF is common, data are scarce on HIV and hemorrhagic fever virus co-infection, such as was the case with our Patient 4. She was also infected with hepatitis B virus. A 68 year old Sierra Leonean man with a history of HIV infection and chronic progressive neurological deterioration was infected with Lassa virus in 2006 [18] The patient survived despite severe disease requiring intubation and mechanical ventilation. In the 2000–2001 outbreak of Ebola virus in Uganda, the CFR was not statistically different between those who were HIV positive and negative [19]. The samples were anonymously tested and no clinical data were reported. Although the clinical data on Patient 4 are also sparse, there were no obvious differences in the clinical manifestations of LHF in this patient compared to the others, with the exception of the aforementioned absence of rash. It is also interesting to note that her peak fever (38.5°C) and leukocyte count (14×10^9^/L) were not particularly high, consistent with her compromised immune system.

There have been very few controlled studies on the management of VHF. Most recommendations represent the informal consensus of experienced clinicians and investigators. Supportive therapy is the mainstay [20]. The pathogenesis of severe cases of VHF is thought to be similar to severe sepsis, with a severe inflammatory response syndrome mediated in part by various soluble cytokines and chemokines and nitric oxide [21]. Therefore, the basic management principles of shock are also recommended for VHF [20], [22] However, since most VHFs occur in resource-poor areas with little access to advanced ICU medicine, opportunities to use and make observations on the efficacy of these or other advanced treatment options are rare.

Although obviously not a controlled trial, we were nevertheless able to make some detailed observations on the management of five patients with LHF, who were often treated in more advanced healthcare settings. The most detailed data are from Patient 5, who was the only patient for whom a specific diagnosis of VHF was considered and confirmed early in the course of disease. Despite receiving ribavirin at disease onset, Patient 5's clinical status deteriorated and her illness was severe and prolonged. Although these results could be interpreted as lack of efficacy of ribavirin against Lujo virus, this is unlikely considering the drug's proven efficacy in other arenavirus infections [8], [23]–[25] Of greater importance was probably the fact that ribavirin was administered orally for the first 6 days of treatment. Efficacy of oral ribavirin for arenavirus infection has not been definitively shown and, in light of the significant first-pass hepatic metabolism resulting in an oral bioavailability of only ∼50%, it is unlikely that oral administration reliably reaches the minimum inhibitory concentration for arenaviruses in serum [26] Serum levels are undoubtedly further diminished by decreased gut absorption, vomiting, and diarrhea in these severely ill patients.

Various adjunctive therapies with demonstrated or theoretical efficacy in severe sepsis were administered to Patient 5 and a few of the other patients, including HMG-CoA reductase inhibitors (statins), N-acetylcysteine [27], [28], recombinant factor VIIa, [29], [30], [31] mechanical ventilation, plasmapheresis, and hemodialysis. Animal models of sepsis have suggested that statin drugs may improve outcomes in septic shock [32], [10]. Furthermore, a large, population-based cohort analysis in Canada showed reduced risk of sepsis in patients with cardiovascular disease who were treated with statins [11]. Patient enrolment is currently ongoing for prospective trials of statin therapy after the development of sepsis. N-acetylcysteine is an antioxidant and free radical scavenger that resulted in decreased nuclear factor-κB and interleukin-8 in patients with sepsis, suggesting a blunting of the inflammatory response [28]. Recombinant factor VIIa is a prohaemostatic agent thought to act at the local site of tissue injury and vascular wall disruption by binding to exposed tissue factor to promote generation of thrombin and platelet activation. [29]. The drug has been used in hemophilia and other coagulation disorders, as well as in liver disease, reversal of anticoagulant therapy, and for episodes of excessive or life threatening bleeding related to surgery or trauma [30], [31]. Other therapies being explored for sepsis and, in some cases specifically for VHF, such as the recombinant inhibitor of the tissue factor/factor VIIa coagulation pathway, rNAPc2, and activated protein C, were not used in this outbreak due to lack of availability and/or risk of bleeding. The seemingly counterintuitive use of anticoagulants like rNAPc2 stemmed from work with an Ebola virus animal model to ameliorate the effects of tissue factor resulting in DIC [21].

It is difficult to assess the contribution of the various therapies to the patient outcomes. Although hemofiltration has been suggested in patients with refractory hemodynamic septic shock, with a significant decrease in ICU mortality in responders [33], and plasmapheresis appeared to have a brief positive effect in Patient 2, we are reluctant to advocate treatments or procedures that potentially increase healthcare worker exposure to blood. In fact, one explanation for the high secondary attack rate associated with this outbreak could be that such high-risk procedures were frequently undertaken.

Many of the drugs employed in the management of Patient 5 are already clinically approved. Investigation of many of these compounds in animal models of VHF is warranted, including in LHF model using strain 13/N guinea pigs [34]. Ideally, controlled clinical trials in humans would also be undertaken, although the feasibility of this is dubious for most VHFs, with the possible exception of Lassa fever, for which many infections occur across West Africa, or perhaps through a “multicenter” approach through advanced planning with Ministries of Health and other partners in endemic areas for VHFs [35], [36], [21]. Until controlled efficacy data are available, and considering the high CFR often associated with VHF, we feel that it is both logical and ethical to explore the use of these approved compounds in treatment of patients with VHF when possible. Treating clinicians should make a concerted effort to collect and publish detailed, repeated, and systematic clinical observations to facilitate objective evaluation of their efficacy.

The pace of discovery of arenaviruses has increased considerably in recent years, with over ten new viruses being isolated since 2000. Pathogenic arenaviruses will almost certainly continue to be discovered. Furthermore, rapid population growth, especially in Africa, and incursion for both economic and leisure activities into natural habitats harboring rodents will likely put humans at risk. The clinical findings and management experience reported here will be of use to clinicians faced with patients with arenavirus infections and as well as other VHFs.

## Supporting Information

Checklist S1 STROBE checklist.
(PDF)
